# Management of spinal-induced hypotension for elective caesarean section: A survey of practices among anesthesiologists from a developing country

**DOI:** 10.4314/ahs.v20i4.50

**Published:** 2020-12

**Authors:** Samina Ismail, Muhammad Sohaib, Fatima Farrukh

**Affiliations:** 1 Aga Khan University Hospital, Anaesthesiology; 2 Aga Khan University, Medical College

**Keywords:** Spinal anesthesia, Hypotension, Cesarean delivery, Vasopressors

## Abstract

**Background:**

In developing countries, more than half of the anesthesia-related maternal deaths are related to spinal hypotension.

**Objective:**

To explore the practices of management of spinal induced hypotension with respect to fluid and vasopressor administration among anesthesiologists from a developing country.

**Methods:**

After approval from institutional ethics committee, an online questionnaire was sent to anesthesiologists registered with Pakistan Society of Anesthesiologists between July and August 2018 to determine management strategies for prevention and treatment of spinal-induced hypotension.

**Results:**

The response rate was 36% (156/433), majority from academic institution (62.8%) with equal representation from attending and trainee anesthesiologist. For prophylaxis 39.1% respondents did not use vasopressors, 32.7% used fluid preloading with crystalloids (54.7%) as fluid of choice followed by combination of co-loading and vasopressor(22.4%). Phenylephrine was the vasopressor of choice for both prophylaxis (33.1%) and treatment (57%). Attending anesthesiologist used a combination of fluid co-loading and vasopressors for prophylaxis as compared to trainee anesthesiologists (37.2% vs. 17.9%; P=0.035) and selected vasopressors according to patient's heart rate (33.3% vs. 19.5%; p=0.05). Prophylactic phenylephrine was used more by respondents from the academic institution (p=0.023). Fluid co-loading was used more by respondents with <30 % compared to those with > 30% of clinical responsibility to obstetric anesthesia (P<0.05).

**Conclusion:**

Phenylephrine as the vasopressor of choice indicates growing awareness of management strategies among anesthesiologists from developing countries but there is a need to increase its use for prophylaxis. Some variation in practice according to the level of anesthesiologist, practice type and responsibilities to obstetric anesthesia are evident.

## Introduction

Obstetric patients develop more extensive block following spinal anesthesia than non-pregnant patients.^1^ As a result, hypotension following spinal anesthesia is a common problem among obstetric patients and has remained a focus of research and controversy for decades. Recent advances have created a better understanding of hemodynamics and choice of vasopressors for prevention and treatment of spinal induced hypotension.^2^, ^3^

Researches in this field have led to the development of a recipe for prevention and management of hypotension following obstetric spinal anesthesia advocating phenylephrine as the first-line vasopressor.^4^, ^5^ Unfortunately, research advances in the developed world have not been translated into practical guidelines to reduce the unacceptable high maternal mortality rate present in resource-limited clinical settings. This is evident from the sixth report on the confidential enquiries into maternal deaths in South Africa where more than half of the anesthesia-related deaths were related to spinal hypotension, and almost all could have been prevented.^6^ Considering high mortality from spinal hypotension, there is still a paucity of literature from developing countries. Most of the surveys on the practices of management of spinal induced hypotension in obstetric patients have been conducted in developed countries.^7^,^8^ In order to bring improvement in resource limited countries, it is important to distinguish between differing clinical contexts as there are marked differences in the availability of staff, training, equipment, drugs and infrastructure between developing and developed countries. Various methods including pharmacological treatment with vasopressors and fluid therapy are used for prevention and treatment of spinal induced hypotension. In addition non-pharmacological methods like use of lateral table tilt or hip elevation with a wedge are employed to prevent hypotension in parturient. 9 The rationale of this study is to find out if the practices of anesthesiologists from a developing country for managing spinal induced hypotension with respect to fluid and vasopressor administration are in accordance with the current recommendation or not.

**Objectives:** The primary objective of this study is to explore the practices of anesthesiologists, from a developing country for prevention and treatment of spinal-induced hypotension with respect to fluid and vasopressor administration, for patients undergoing elective caesarean section (CS) .This, in future, can be instrumental in the development of clinical guidelines in the context of developing countries.

## Methods

**Design:** In order to conduct this prospective survey, a questionnaire was initially developed by two attending anesthesiologists practicing obstetric anesthesia; after reviewing surveys and publications on the management of spinal induced hypotension in obstetric patients.^8^ In order to check the internal validity of the questionnaire and its use in the local context of a developing country, the questionnaire was distributed to four attending anesthesiologists, two from an academic institution and two from a non-academic private practice. In addition, the questionnaire was distributed to four trainee residents working in an academic institution. After one week, the same anesthesiologists were asked to fill the same questionnaire (excluding the demographics) and it was observed that 87% of the questions were answered in a similar manner on the second occasion.

To ensure confidentiality, survey responses did not contain any personal identifying information. Email addresses were used solely by the principal investigator to ensure there was no duplication of the questionnaire forms. The survey questionnaire gives a brief background of the study and consent for the study. This is followed by anesthesiologist's demographic data and routine methods for preventing and treating spinal-induced hypotension with an emphasis on fluid administration and vasopressor use. The choices for the prevention of spinal-induced hypotension following elective cesarean delivery included the use of fluid preloading or co-loading as the sole technique or in combination with vasopressors, vasopressors only, or none of the above.

A fluid preload was defined as a rapid intravenous (IV) fluid bolus starting before the induction of spinal anesthesia and continuing this fluid during the performance of the block. A fluid co-load was defined as a rapid IV fluid bolus starting either during or immediately after the induction of spinal anesthesia. Influence of maternal heart rate on choice of vasopressors, and thresholds for treating hypotension and bradycardia was also explored. Respondents were given the opportunity to submit additional comments.

**Settings:** After getting approval from the institutional ethics committee, an online survey form was uploaded on Google Forms and remained open from 2nd July to 18th August, 2018. An email with an explanation of the purpose of the study with a link to the online survey form was sent to the participants. Non-responders were emailed twice until 18^th^ August 2018. The survey responses, obtained via email, were stored on a Microsoft Excel sheet and then exported to SPSS for analysis.

**Participants:** Anesthesiologists registered with Pakistan Society of Anesthesiologists (PSA) both attending and trainees working either in academic institutions, private settings or both.

**Main outcome measures:** Anesthesiologists' choice for preventing and treating spinal-induced hypotension with an emphasis on fluid administration and vasopressor use.

**Statistical analysis:** Data were analyzed by statistical packages for social science version 19 (SPSS Inc., Chicago, IL). Frequency and percentage were computed for qualitative observations. Chi-square test or Fisher exact test was used to compare method of prevention, type of fluids to prevent spinal induced hypotension, routine vasopressors for prophylaxis and routine vasopressor(s) for the treatment between level of anesthesiology, clinical responsibility (<30% vs. ≥30%) and type of practice at p≤ 0.05 level of significant.

## Results

Four hundred and thirty three anesthesiologists registered with PSA were contacted via email and 156 responded, representing an overall response rate of 36% (156/433). Responses were received from all four provinces of Pakistan, majority from the province of Sind (59%, n=92) followed by Punjab (33.3%, n=52), Khyber Pakhtunkwa (3.8%, n=6) and Baluchistan (3.8%, n=6). The responses according to the level of anesthesiologists (attending or trainee anesthesiologists) were equal. Responses from anesthesiologists having obstetric anesthesia responsibility of >30% of their clinical workload was 35.9 %(n=56).

**Figure 1:**
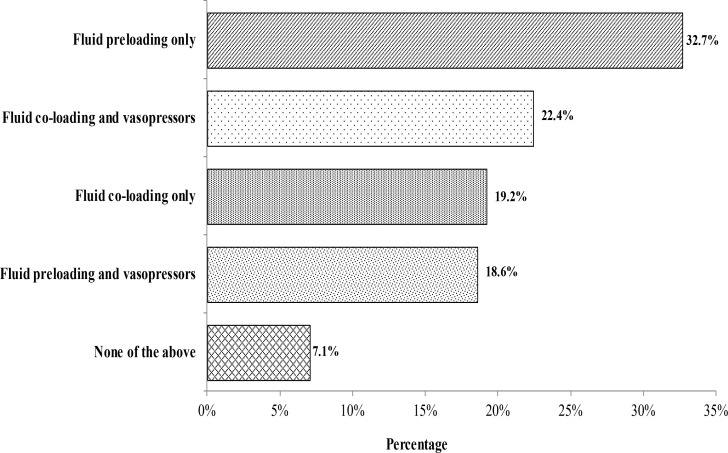
Methods routinely used to prevent spinal induced hypotension (n=156)

The methods used to prevent spinal induced hypotension are shown in Figure 1. The most common methods used were fluid pre-loading followed by combination of fluid co-loading and vasopressor. Majority of the participants were using only crystalloids (54.7%, n=85) as fluid of choice. Other choices of fluids were combination of crystalloids and colloids (28.8%, n=45) and colloids (10.3%, n=16). Regarding the volume for prophylaxis 0.5–1 L was chosen by 54.5% (n=85) of the respondents, while 9.6% (n=15) used between 1–2 L, and 1.3% in excess of 2 L.

**Figure 2:**
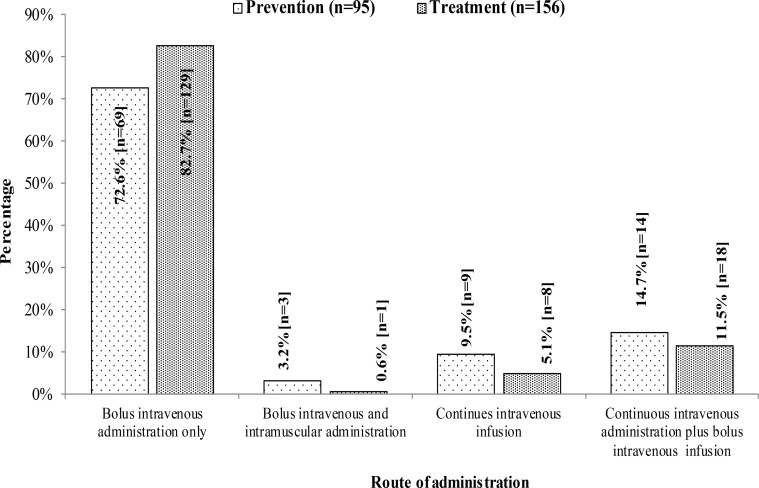
Route of administration of vasopressors for the prevention and treatment of spinal-induced hypotension

The choice of vasopressor for the prevention of spinal hypotension is shown in table 1. Phenylephrine was the choice in majority (33.1%) followed by choosing vasopressor according to patients' heart rate (19.9%). The route of administration for prophylactic vasopressor as shown in figure 2 is indicating that intravenous bolus administration was the most popular method. There were 39.1% (n=61) participants who did not use any prophylactic vasopressors for prevention of spinal induced hypotension.

**Table 1:** Choice of vasopressors for the prevention and treatment of Spinal-induced hypotension

Choice of vasopressors	Prevention n= 95	Treatment n=156
Ephedrine	8(5.1)	13 (8.3)
Phenylephrine	52(33.1)	89(57)
Phenylephrine and ephedrine administered together	2(1.3)	1(0.6)
Either phenylephrine or ephedrine based on the patients' heart rate	31(19.9)	41(26.3)
Epinephrine	1(0.6)	7 (4.4)
Norepinephrine	1(0.6)	5(3.2)

Data are numbers (%)

The choice of vasopressor for the treatment of spinal hypotension was phenylephrine (57%) and the second most popular approach in 26.3% of participants was either phenylephrine or ephedrine based on patient's heart rate (Table 1). The choice of route of administration of vasopressors for the treatment of spinal induced hypotension was intravenous bolus in 82.7% (n= 129) of participants. (Figure 2)

The trigger heart rate for not choosing phenylephrine (or other alpha agonists) for the treatment of spinal hypotension was heart rate <60 beats /minute among 66.7% (n=104) of participants and <80 beats /min in 23.3% (n=37) of participants.

The most common threshold for treating spinal induced hypotension was either an absolute fall or fall in percentage of either systolic or mean blood pressure. When considering the fall in the percentage of blood pressure, 58.3% (n=91) of participants took a fall of <20% of systolic blood pressure as a trigger for treating spinal induced hypotension, 11.5% (n=18) took a fall <25% and 10.3% (n=16) a fall of <10% of systolic blood pressure. There were 7.1% (n=11) of participants who aimed to maintain blood pressure at baseline and any fall less than baseline was a trigger for treatment.

When considering the fall in absolute value of blood pressure, 41.7%(n=65) of participants used treatment when systolic blood pressure fell below 100 mmHg, 32.1% when it fell below 90 mmHg and 20.5% (n=32) when it fell below 80mmHg. Among participants who used absolute value of mean arterial pressure as the trigger of treatment; 37.8% (n=59) used <60 mm Hg, 28.2% (n=44) used <65 mmHg and 32.1% (n=52) used <50 mmHg as a trigger for the treatment of spinal hypotension.

The effect of the level of anesthesiologists (attending or trainee) on the choice for the management of spinal induced hypotension is shown in Table 2 and Table 3. In the analysis, the level of anesthesiologists influenced the choice of methods of prevention and choice of vasopressors for treatment of spinal induced hypotension. Attending anesthesiologist used a combination of fluid co-loading and vasopressors for prophylaxis as compared to the trainee anesthesiologists (37.2% vs. 17.9%; P=0.035). In addition, a statistically significant higher number of attending anesthesiologists compared to trainee anesthesiologists based the choice of vasopressors according to the patient's heart rate for treatment of spinal induced hypotension (33.3% vs. 19.5%; p=0.05).

**Table 2:** Effect of level of anesthesiologists (attending vs. trainee), clinical responsibility to obstetric anesthesia and type of practice (academic vs. private vs. both) on methods and types of fluids for prevention of spinal induced hypotension

	Number	Level of anesthesiologist		P-Value	Clinical responsibility		P-Value	Type of practice			P-Value
			
		Attending	Training		≥30%	<30%		Academic Institution (AI)	Private Institution (PI)	Both	
**Methods of** **Prevention**		**n=78**	**n=78**		**n=56**	**n=100**		**n=98**	**n=13**	**n=45**	
Fluid preloading only	51	21(26.9%)	30(38.5%)	0.125	19(33.9%)	32(32%)	0.805	29(29.6%)	6(46.2%)	16(35.6%)	0.435
Fluid preloading and vasopressors	29	16(20.5%)	13(16.7%)	0.537	14(25%)	15(15%)	0.124	23(23.5%)	3(23.1%)	3(6.7%)	0.051
Fluid coloading only	30	16(20.5%)	14(17.9%)	0.685	5(8.9%)	25(25%)	0.015	20(20.4%)	1(7.7%)	9(20%)	0.544
Fluid coloading and vasopressors	35	23(29.5%)	12(15.4%)	0.035	18(32.1%)	17(17%)	0.03	20(20.4%)	2(15.4%)	13(28.9%)	0.432
None of the above	11	2(2.6%)	9(11.5%)	0.029	0(0%)	11(11%)	0.008	6(6.1%)	1(7.7%)	4(8.9%)	0.832
**Type of fluids** **to prevent** **spinal** **induced** **hypotension?**		**n=78**	**n=75**		**n=56**	**n=97**		**n=96**	**n=13**	**n=44**	
Crystalloids only	85	43(55.1%)	42(56%)	0.914	28(50%)	57(58.8%)	0.293	61(63.5%)	7(53.8%)	17(38.6%)	**0.022**
Colloids only	16	9(11.5%)	7(9.3%)	0.656	7(12.5%)	9(9.3%)	0.53	7(7.3%)	1(7.7%)	8(18.2%)	0.14
Both crystalloids and colloids	45	25(32.1%)	20(26.7%)	0.465	21(37.5%)	24(24.7%)	0.095	25(26%)	4(30.8%)	16(36.4%)	0.458
None of the above	7	1(1.3%)	6(8%)	0.06	0(0%)	7(7.2%)	0.048	3(3.1%)	1(7.7%)	3(6.8%)	0.533

**Table 3:** Effect of level of anesthesiologists (attending vs. trainee), clinical responsibility to obstetric anesthesia and type of practice (academic vs. private vs. both) on the routine use of vasopressors for prophylaxis and treatment

	Number	Level of anesthesiologist		P-Value	Clinical responsibility		P-Value	Type of practice			P-Value
			
		Attending	Training		≥30%	<30%		Academic Institution (AI)	Private Institution (PI)	Both	
**Routine vasopressors** **for Prophylaxis**		**n=78**	**n=78**		**n=56**	**n=100**		**n=98**	**n=13**	**n=45**	
Ephedrine	8	5(6.4%)	3(3.8%)	0.468	4(7.1%)	4(4%)	0.459	2(2%)	2(15.4%)	4(8.9%)	**0.049**
Phenylephrine	52	24(30.8%)	28(35.9%)	0.497	18(32.1%)	34(34%)	0.813	37(37.8%)	3(23.1%)	12(26.7%)	0.305
Phenylephrine and ephedrine administered together	2	2(2.6%)	0(0%)	0.497	1(1.8%)	1(1%)	0.999	1(1%)	1(7.7%)	0(0%)	0.088
Either phenylephrine or ephedrine based on the patients' heart rate	31	17(21.8%)	14(17.9%)	0.548	15(26.8%)	16(16%)	0.105	24(24.5%)	3(23.1%)	4(8.9%)	0.09
Epinephrine	1	0(0%)	1(1.3%)	0.999	0(0%)	1(1%)	0.999	1(1%)	0	0	0.742
Norepinephrine	1	1(1.3%)	0(0%)	0.999	0(0%)	1(1%)	0.999	1(1%)	0	0	0.742
Do not use prophylaxis vasopressors	61	29(37.2%)	32(41%)	0.623	18(32.1%)	43(43%)	0.183	32(32.7%)	4(30.8%)	25(55.6%)	**0.027**
**Routine** **vasopressor(s)** **for Treatment**		**n=78**	**n=77**		**n=56**	**n=99**		**n=97**	**n=13**	**n=45**	
Ephedrine	13	9(11.5%)	4(5.2%)	0.154	7(12.5%)	6(6.1%)	0.227	8(8.2%)	3(23.1%)	2(4.4%)	0.102
Phenylephrine	89	40(51.3%)	49(63.6%)	0.12	26(46.4%)	63(63.6%)	0.037	61(62.9%)	3(23.1%)	25(55.6%)	**0.023**
Phenylephrine and ephedrine administered together	1	1(1.3%)	0(0%)	0.999	1(1.8%)	0(0%)	0.361	1(1%)	0	0	0.74
Either phenylephrine or ephedrine based on the patients' heart rate	41	26(33.3%)	15(19.5%)	0.051	19(33.9%)	22(22.2%)	0.112	23(23.7%)	5(38.5%)	13(28.9%)	0.478
Epinephrine	3	0(%)	3(3.9%)	0.12	2(3.6%)	1(1%)	0.266	2(2.1%)	0	0	0.868
Norepinephrine	1	1(1.3%)	0(0%)	0.999	0(0%)	1(1%)	0.999	1(1%)	0	0	0.74
Non and Other	7	1(1.3%)	6(7.8%)	0.063	1(1.8%)	6(6.1%)	0.423	1(1%)	2(15.4%)	4(8.9%)	**0.016**

Regarding the type of practices; most of the participants were working in academic institutions (62.2%). The numbers of participants were least from private practice (8.3%); however, 28.3% of the participants worked both in academic institutions and also had their private practice. On comparing all three practices, statistically significant difference was found in the method and type of fluid for prevention and choice of vasopressors for the treatment of spinal induced hypotension. Crystalloids for prophylaxis were used as the fluid of choice by the respondents from the academic institution. Phenylephrine for prophylaxis and treatment was the choice of vasopressors among respondents from the academic institution compared to respondents from other two practices. However the difference was statically significant for treatment (p=0.023) but not for prophylaxis among the three practices. The practice of not using prophylactic vasopressors was found more among anesthesiologists working both in private and academic institutions compared to working only in private institution (p=0.027).

The results of this study showed that practice of prevention and treatment of spinal induced hypotension was different among respondents having less or more than 30% clinical responsibility to obstetric anesthesia. For prevention of spinal induced hypotension, respondents with >30% responsibility to obstetric anesthesia used a combination of fluid co-loading and vasopressors as compared to only fluid co-loading by respondents with <30 % of clinical responsibility to obstetric anesthesia (P<0.05). In addition phenylephrine was the choice of vasopressor among respondents with <30% clinical responsibility to obstetric anesthesia, however more respondents with >30% of clinical responsibility selected vasopressors according to patient's heart rate although that was not statistically significant.

## Discussion

Considering the paucity of literature in developing countries, this study evaluates the practices of anesthesiologists from a developing country for prevention and treatment of spinal hypotension, in patients scheduled for elective CS. This survey also highlights the variations in practices among attending and trainee anesthesiologists, among those practicing in academic institutions compared to those in private practice and between anesthesiologist having >30 % of their workload in obstetric anesthesia compared to those having less exposure to obstetric anesthesia.

Recommended strategies for preventing and treating obstetric spinal induced hypotension employ a combination of fluid and vasopressor with phenylephrine as the first line agent. 4, 5, 10, 11 The result of this study reveals that the participants' choice for vasopressor is in congruence with current research, as phenylephrine was the overall choice of prophylaxis in 33.3% and for treatment of spinal hypotension in 57.1% of our participants. In addition, 22.4% of participants are using a combination of co-loading and vasopressors.

However, recent literature has moved the debate from the choice between ephedrine and phenylephrine to the manner in which phenylephrine should be given. Investigations with lower-dose phenylephrine infusions support prophylactic infusions in a range of 25 – 50 µg/min as part of routine CS as they give the most benefit with the fewest side-effects. 12–14 One systematic review concluded that phenylephrine infusions given prophylactically caused reduction in the incidence of maternal hypotension, nausea and vomiting without affecting other important maternal or neonatal outcomes.^15^ In the current study, it was observed that a significant percentage of anesthesiologists' current practices regarding prophylaxis for spinal hypotension is not according to the recent recommendation as 39% of the respondents did not use any kind of prophylactic vasopressor, and 33.3% are still using fluid preloading. However, the results of this study are not different from the survey of the anesthetic practices of the Society for Obstetric Anaesthesia and Perinatology (SOAP) members by Duke University's Department of Anesthesiology, where 33% of the participants are still using fluid preloading only 21% are using fluid co-loading and vasopressors for prophylaxis. 8 However, this survey was done a decade ago and how valid the comparison is with the current practice of SOAP members cannot be commented upon.

It is evident that despite overwhelming evidence for the benefit of prophylactic phenylephrine infusions in elective patients, clinicians are reluctant to implement these findings even in resource-rich settings. This is evident from the surveys done in United Kingdom (UK) and other European countries, 7, 16 indicating that vasopressors are not preferred for prophylaxis and ephedrine is still the vasopressor of choice for the management of obstetric spinal hypotension among participating anesthesiologists.

The possible reason could be that there is no mention of prophylactic vasopressor infusions or phenylephrine as the vasopressor of choice for caesarean section in National Institute for Health and Care Excellence (NICE) clinical guidelines and other UK based guidelines.^17^, ^18^ In addition, in developed world choice of vasopressor was perceived as ‘not being quite a life and death issue’,^19^ however this is critical in limited resource areas, where spinal hypotension may contribute to more than half the anesthesia related deaths.^6^ South Africa is one of the few developing countries that routinely collects and analyses the data through confidential enquiry process.^19^ In their 2011–2013 report, more than half of the South African anesthesia related deaths were related to spinal hypotension and, in 7% of these cases, there was no health care worker designated solely to provide anesthesia services. 6 Therefore, effective strategies in developing countries should consider an emphasis on preventative rather than reactive management. Due to lack of resources, one person is fulfilling more than one job, and senior anesthesiologists who can promptly pick up signs of hypotension and do reactive management may not be available in remote areas of a developing country.

This study went further and evaluated the difference of practice between trainees and attending anesthesiologists. The results revealed a significant difference in the method used for prophylaxis and treatment of spinal hypotension. Attending anesthesiologists are using a combination of fluid co-loading and vasopressors for prophylaxis and choosing vasopressors according to the patient's heart rate for treatment of spinal hypotension. In addition a significantly higher number of anesthesiologists with >30% clinical responsibility to obstetric anesthesia were also using a combination fluid co-loading and vasopressors for prophylaxis of spinal hypotension. This shows that experience gives an insight to base the practice according to the current recommendations. In addition, this study observed that significantly higher numbers of respondents from purely academic institution were using a combination of vasopressors with fluid for prophylaxis and phenylephrine was the choice of vasopressors for treatment of spinal induced hypotension. However compared to a previous survey, 8 this study could not clearly evaluate the difference based on practice (academic vs. private) as in this survey only 8.3% of the participants were working purely in private set up. The rest of the participants were either from an academic institution (62.8%) or working both in an academic institution and having their private practice in the evening (28.8%).

One limitation of this survey is a low response rate of 36% which, although within published findings of 25–30%, could have been improved by using a mixed-mode approach which combines both mailed and e-mailed survey instruments with an Internet-based response mechanism.^20^, ^21^. Another limitation is unequal representation of participating anesthesiologist from four provinces of Pakistan. Survey responses were not collected equitably from Pakistan's provinces as a majority of the participants practice in Sindh (59%) and Punjab (33.3%), whereas only 1.8% participants practice in Baluchistan and another 1.8% in Khyber Pakhtunkwa. The possible reason could be less familiarity with computers and Google survey forms in two less developed provinces of Pakistan. In addition, 73% of Pakistan population is from Sindh and Punjab with Baluchistan and Khyber Pakhtunkwa representing only 7% and 20% of Pakistan's population respectively. 22

## Conclusion

This survey highlights the increasing use of phenylephrine as the drug of choice for treatment of spinal anesthesia induced hypotension, however its use for prophylaxis is less. There is some variation in practice according to the level of anesthesiologist, practice type and responsibilities to obstetric anesthesia. There is not a wide variation of practices of managing spinal induced hypotension from the developed world, showing growing awareness regarding management strategies among anesthesiologist from developing countries. However, considering the high mortality of parturient from spinal induced hypotension, preventive strategies are more important in resource limited settings especially amongst anesthesiologists working in a setting where they have more than one responsibility to fulfill. It is therefore recommended that developing countries should collect their own data and assess their available resources to formulate practical guidelines based on current research and internationally accepted management protocols.
